# A novel form of constitutively active farnesylated Akt1 prevents mammary epithelial cells from anoikis and suppresses chemotherapy-induced apoptosis

**DOI:** 10.1038/sj.bjc.6600566

**Published:** 2002-10-07

**Authors:** M Schmidt, S Hövelmann, T L Beckers

**Affiliations:** ASTA Medica Oncology, Weismüllerstr. 45, D-60314 Frankfurt, Germany

**Keywords:** protein kinase B/Akt, apoptosis, chemoresistance, Anoikis

## Abstract

Protein kinase B/Akt has been described as a central mediator of anti-apoptotic signals transduced by the PI3 kinase. Although the role of Akt in the suppression of apoptosis is well elucidated, a potential function of Akt in tumorigenesis and chemoresistance is less intensively documented. In this study, we describe the construction of a novel form of constitutively active Akt1, which relies on the deletion of its pleckstrin homology domain and the insertion of a C-terminal farnesylation sequence. Stable cell lines were generated with MCF10A mammary epithelial cells and A549 human NSCLC cells expressing constitutively active Akt1. Enigneered MCF10A cells were rendered resistant towards apoptosis resulting from loss of cellular substrate attachment (anoikis). We investigated the chemosensitivity of A549 cells expressing farnesylated Akt *vs* control cells. A profoundly decreased sensitivity towards Mitoxantrone and cisplatin was observed in cells expressing farnesylated Akt. No significant difference in sensitivity however was observed upon treatment with cell cycle specific chemotherapeutic agents like paclitaxel. Our data suggest, that Akt is a central mediator in the suppression of anoikis and modulation of chemotherapy-induced apoptosis. Therefore it represents a promising target for small molecule inhibitors to shift the apoptotic threshold in cancer cells after treatment with standard chemotherapy.

*British Journal of Cancer* (2002) **87**, 924–932. doi:10.1038/sj.bjc.6600566 www.bjcancer.com

© 2002 Cancer Research UK

## 

A prerequisite of transformed cells in order to form solid tumours is the acquisition of growth properties that allow them to divide indefinitely and in an uncontrolled fashion. During the process of malignant transformation which requires molecular alterations like activation of proto-oncogenes, and suppression of tumour-suppressor genes, tumour cells have to thrive under conditions of low oxygen and poor nutrient supply. Such conditions would normally lead to the onset of programmed cell death to eliminate potentially dangerous cells from the organism. Tumour cells must therefore have acquired molecular mechanisms to counteract apoptotic triggers in order to survive under unfavourable conditions and to be able to form solid tumours. It is assumed that mechanisms responsible for anti-apoptotic signalling might be equally important for the development of the pathological cancer phenotype as proliferative signalling cascades.

One pathway that has been shown to mediate important survival signals for mammalian cells comprise receptor tyrosine kinases like PDGFR or IGF-1R (Franke *et al*, 1995; Kohn *et al*, 1995) that activate the PI3 kinase upon binding of their respective ligands. Activation of phosphatidyl-inositol 3-kinase (PI3K) protects cells from apoptosis and is hence considered a survival signal. The phosphatidylinositide lipids produced by PI3K can stimulate multiple kinases, including protein kinase B/Akt, which is a central downstream mediator of anti-apoptotic signals transmitted by PI3K (Datta *et al*, 1999). The amino terminus of Akt contains a PH domain that recruits the protein to the cell membrane by binding to the PI3K product PI(3, 4, 5)P_3_ and PI(3, 4,)P_2_ leading to a conformational change (Alessi and Cohen, 1998). This conformational change allows Akt to be phosphorylated at threonine 308 and serine 473 by the kinases PDK1 and a postulated kinase PDK2. A number of potential substrates within the apoptotic machinery have been identified that are phosphorylated by Akt within the consensus sequence RXRXXS/T (Obata *et al*, 2000). The first Akt substrate identified was glycogen synthase kinase 3 (GSK3) (Cross *et al*, 1995). Phosphorylation of GSK3 at serine 9 results in its inactivation. Besides regulating glycogen synthesis, GSK3 is involved in the regulation of several intracellular signalling pathways including AP1 (Scalia *et al*, 2001), CREB (Cross *et al*, 1995), and the tumour suppressor gene product APC (Zumbrunn *et al*, 2001). Phosphorylation of the pro-apoptotic protein Bad at serine 136 by Akt creates a binding site for 14-3-3 proteins and thereby prevents Bad from binding and inhibiting the anti-apoptotic protein Bcl-x_L_ (Datta *et al*, 1997). Similarily, phosphorylation of the forkhead transcription factor FKHRL1 by Akt at threonine 32 and serine 253 creates a binding motif for 14-3-3 proteins resulting in cytoplasmic retention of FKHRL1 (Brunet *et al*, 1999). Transcriptional targets of protein kinase B/Akt (PKB/Akt) include the pro-apoptotic proteins like Fas resulting in transcriptional downregulation dependent on activated Akt (Peli *et al*, 1999). Caspases are intracellular proteases that function as initiators and effectors of apoptosis. Akt directly phosphorylates caspase-9 at serine 196 and inhibits its protease activity (Cardone *et al*, 1998). Activation of the NF-κB pathway by Akt has been demonstrated by the direct association of Akt with IKK. *In vitro* phosphorylation of IKK by Akt is supposed to enhance the degradation of IκB and consequently the translocation of NF-κB into the nucleus (Ozes *et al*, 1999). However, results from these *in vitro* kinase assay have not been confirmed (Delhase *et al*, 2000). Other substrates of Akt include apoptosis signal-regulating kinase 1 (Kim *et al*, 2001), phosphofructokinase 2 (Deprez *et al*, 1997), Mdm2 (Mayo and Donner, 2001) and endothelial NO synthase (Dimmeler *et al*, 1999). The dominant negative caspase-8 homologue c-FLIP is upregulated in tumour cell lines on a transcriptional level by the PKB pathway through a yet unknown mechanism (Panka *et al*, 2001). *In vivo*, targeted disruption of Akt2 leads to insulin resistance and mild diabetes mellitus-like syndrome (Cho *et al*, 2001a). The metabolic abnormalities resulting from Akt2 deficiency could not be compensated by Akt1 or Akt3, reflecting differences in the substrate specificity, relative abundance in insulin-responsive tissues, or both. Non redundant functions of Akt isoforms were further emphasised by targeted deletion of Akt1. These animals display a conspicuous impairment in organismal growth, but no defects in glucose meatbolism (Cho *et al*, 2001b).

Besides playing a role in insulin signalling and in mediating anti-apoptotic stimuli triggered by multiple exogenous stimuli, Akt has been shown to be overexpressed in a variety of human tumour cell lines and cancers (Bellacosa *et al*, 1995; Ringel *et al*, 2001; Roy *et al*, 2002) and to be a mediator of oestrogen resistance in breast cancer cells (Campbell *et al*, 2001). Moreover, Akt activity can be deregulated by constitutive activation or by inhibition of PTEN/MMAC1, a phosphatase that directly counteracts Akt through the dephosphorylation of PI-3-, 4-, 5-P_3_ (Stambolic *et al*, 1998). Overexpression of activated Akt can suppress anoikis, which is the induction of apoptosis after detachment of epithelial cells from extracellular matrix (Rytömaa *et al*, 2000).

Due to its potent capacities as modulator of anti-apoptotic tumour cell signalling Akt is discussed as a potential target for small molecule kinase inhibitors. These are expected to shift the apoptotic threshold in cancer cells after treatment with chemotherapy and might be a way in counteracting the effects of loss of the tumour suppressor PTEN, which normally acts to inhibit PKB.

Despite the fact that Akt has been intensively studied in anti-apoptotic signalling and has been shown to be overexpressed in various types of cancer, relatively few data support the role of Akt in downregulating the sensitivity of cancer cells towards standard chemotherapy (Page *et al*, 2000; Hayakawa *et al*, 2000). To further explore the role of Akt in modulating chemotherapy or matrix-detachement-induced apoptosis, we designed a novel form of a constitutively active Akt1: we deleted the N-terminal PH domain, which is responsible for membrane attachment and subsequent activation and inserted a C-terminal sequence tag encoding a farnesylation motif and a stretch of basic amino acids to target Akt1 to the membrane. Deletion of the PH domain unmasks the residues mandatory for activation by phosphorylation (Alessi and Cohen, 1998) and the ectopically expressed protein can be released from the membrane by cellular farnesyl esterases. With this Akt1-construct we generated stable cell clones of MCF10A mammary epithelial cells and A549 non small cell lung cancer (NSCLC) cells expressing constitutively active farnesylated (CA) Akt1. Here we describe the effects of CA-Akt on the suppression of anoikis and modulation of chemosensitivity towards a panel of chemotherapeutic agents currently used in the clinic.

## MATERIALS AND METHODS

### Materials

Anti-Akt and anti-phospho-Akt antibodies were obtained from Cell Signalling Technology, Inc. (Beverly, MA, USA); M2 anti-FLAG antibody, β-actin antibody, and all other reagents were purchased from Sigma Chemical (St. Louis, MO, USA) unless otherwise specified.

### Cells and cell culture

A549 human lung cancer cells were obtained from the National Cancer Institute (Frederick, MD, USA) and were maintained in Ham's F12 medium supplemented with 10% FBS, 100 u ml^−1^ penicillin G, and 100 μg ml^−1^ streptomycin. MCF10A human mammary epithelial cells (ATCC) were maintained in DMEM : F12 medium supplemented with 5% horse serum, 0.5 μg ml^−1^ hydrocortisone, 10 μg ml^−1^ insulin, 5 nM EGF, 100 u ml^−1^ penicillin G, and 100 μg ml^−1^ streptomycin.

### Construction of the pcDNA vectors containing farnesylated Akt1 devoid of the PH domain

RNA was prepared from MCF10A cells using the RNeasy kit from Qiagen (Hilden, Germany). Subsequently the RNA was transcribed into cDNA with the first strand cDNA kit from Roche Diagnostics (Indianapolis, IN, USA) using an oligo-dT primer as supplied by the manufacturer. Human Akt1 devoid of its PH domain was amplified by PCR using the forward primer 5′-AAGGACGACGACGACCTGGAATTCGCTGACGGCCTCAAGAA-3′ and the backward primer 5′-CTTGCTCTTCTTCTTCTTCTTCTTTCTAGAGGCGCTGCTGCTGGC-3′. In a second round of PCR the resulting fragment was amplified with the forward primer 5′-ATGCTAGCGCCACCATGGACTACAAGGACGACGACGAC-3′ and the backward primer 5′-TATGTTTAAACTCACATGATCACGCACTTGGTCTTGCTCTTCTTCTT-3′. The final PCR product encoded Akt1 devoid of its PH domain (aa 1–106) with an N-terminal fusion of the FLAG antibody epitope and a C-terminal fusion of a poly-basic tail and a farnesylation sequence. The PCR fragment was subcloned into pcDNA 3.1 Hygro and pcDNA6 vectors via the restriction sites *Nhe*I and *Pme*I included in the polylinker of the vector and the respective PCR primers.

### Establishment of farnesylated Akt1 expression clones in MCF10A and A549 cells

Transfection was performed with the Fugene-6 transfection kit (Roche Diagnostics, Indianapolis, IN, USA) according to the manufacturer's recommendations. Stable MCF10A clones transfected with the pcDNA3.1 Hygro derivative were selected in medium containing 20 μg ml^−1^ Hygromycin B (Roche Diagnostics, Indianapolis, IN, USA), A549 clones transfected with the pcDNA6 derivative were selected in growth medium supplemented with 70 μg ml^−1^ Blasticidin S (Invitrogen, Carlsbad, CA, USA). Stable clones were analysed for expression of ectopic FLAG-tagged Akt1 devoid of its PH domain and containing the farnesylation sequence by immunoblotting with the M2 antibody specific for the FLAG-tag. Clones that were both, resistant towards the respective antibiotic and positive on protein level, were selected for further studies. Clones expressing farnesylated Akt1 were named A549-Akt1 or MCF10A-Akt1, respectively. Experiments were conducted with at least two different clones.

### Cytotoxicity assay

Cells were seeded onto 96-well culture plates and incubated in 100 μl growth medium supplemented with 0.5% FCS. After 24 h the cells were treated with various doses of chemotherapeutic drugs as indicated in 50 μl volume. Seventy-two hours after addition of the drugs cell viability was assayed by adding 75 μl of 1 mg ml^−1^ XTT (sodium 3′-[1-(phenyl amino carbonyl-3,4-tetrazolium]-bis (4-methoxy-6-nitro-) benzene sulphonic acid) in RPMI containing 7.66 μg ml^−1^ PMS (N-methyl dibenzopyrazin methyl sulphate). After incubating the cells for 3 h at 37°C in a CO_2_ incubator, cell viability was then determined by measuring the optical absorbance at a wavelength of 490 nm and normalisation with the corresponding control cells treated with 1% DMSO.

### Immunoblot analysis

Cells were lysed in a lysis buffer containing 50 mM Tris, pH 7.4, 150 mM NaCl, 1% NP-40, 50 mM NaF, 1 mM Na_3_VO_4_, 1 mM phenylmethylsulphonyl fluoride. The lysates were cleared by centrifugation and the supernatants were collected. Equal amounts of lysate protein were used for Western blot analysis with antibodies as indicated. Specific signals were visualised by use of the ECL chemoluminescence detection kit (Amersham, Braunschweig, Germany).

### Determination of Akt1 phosphorylation

Cells were seeded out in 6-well plates and switched to medium containing 0.5% serum after attachment. After starvation overnight the cells were stimulated for 15 min. with medium containing 10% serum or medium additionally supplemented with a cocktail containing each 10 ng ml^−1^ of EGF, PDGF, and IGF-1. Cell lysates were then analysed in immunoblot assays with antibodies from Cell Signaling Technologies (Beverly, MA, USA) specific for phospho-Akt (Thr 308) or phospho-Akt (Ser 473). Signals were visualised by use of the ECL chemoluminescence detection kit (Amersham, Braunschweig, Germany).

### Akt kinase assay

The Akt kinase assay was performed using the Akt Kinase Assay Kit from Cell Signalling Technology, Inc. with GSK-3 fusion protein as substrate. Cells were seeded out in 10 cm dishes and switched to medium containing 0.5% serum after attachment. After starvation overnight the cells were either kept unstimulated or were stimulated for 20 min with medium containing 10% foetal calf serum (FCS) supplemented with a cocktail containing each 10 ng ml^*−*1^ of EGF, PDGF, and IGF-1. After cell lysis and clarification by centrifugation, the lysates were swirled for 3 h at 4°C with an anti Akt-agarose conjugate (control transfected cells) or with an anti M2-Flag-agarose conjugate (cells expressing farnesylated Akt1). After three times washing with lysis buffer and with kinase buffer the immunoprecipitates were subjected to an *in vitro* kinase reaction with 40 μl of reaction mixture containing kinase reaction buffer supplemented with 200 μM ATP and 1 μg GSK-3-fusion protein. The reaction was allowed to process at 30°C for 30 min and stopped by boiling the samples in SDS sample buffer for 5 min; the products were separated by 12.5% SDS–PAGE. Immunoprecipitates were then analysed in immunoblot assays with antibodies specific for phospho-GSK (Ser 21/9) and Akt. Signals were visualised with the the ECL chemoluminescence detection kit (Amersham, Braunschweig, Germany).

### Induction of anoikis

To prevent cell attachment, tissue culture plates were coated twice with a solution of 5 mg ml^−1^ poly-HEMA (Sigma, St. Louis, MO, USA), dried and rinsed with PBS. The cells were subsequently added into the plates in medium as indicated in the experiments and induction of apoptosis through loss of cell attachment was measured after 24 h of incubation at 37°C (Frisch and Francis, 1994).

### Quantitation of apoptosis by ELISA

To detect the onset of apoptosis in the target cells, an apoptosis detection ELISA kit (Roche Diagnostics, Indianapolis, IN, USA) was used according to the manufacturer's instructions. This photometric enzyme immunoassay quantitatively measures cytoplasmic histone-associated DNA fragments (mononucleosomes and oligonucleosomes), that are characteristic of apoptotic cell death. Triplicate aliquots of 10^4^ cells well^−1^ were seeded in 96-well plates and treated as indicated. After incubation for 24 h at 37°C in 5% CO_2_ and 95% humidified air, the apoptosis assay was then carried out according to the manufacturer's instructions to quantify cytoplasmic histone-associated DNA fragments. Optical absorbance was measured at a wavelength of 405 nm in a microplate reader.

## RESULTS

**Figure 1:**
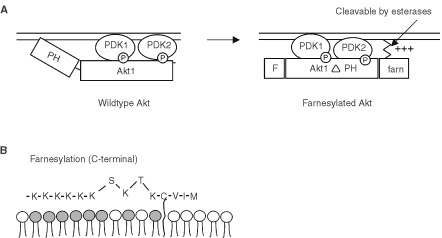
Construction of a farnesylated constitutively active Akt1 expression vector. (**A**) While wildtype Akt1 depends on its PH domain for membrane attachment and subsequent phosphorylation at Thr 308 by PDK1 and Ser 473 by a postulated PDK2 (left), the expression construct described herein is devoid of the PH domain and contains a C-terminal farnesylation tag that is supposed to alleviate membrane dislocation after activation. (**B**) Schematic presentation of the farnesylation sequence including a poly-basic tail for energetically favored membrane attachment. F, sequence encoding the FLAG tag for antibody detection.

To investigate the role of Akt in the modulation of the onset of anoikis and of chemosensitivity in cancer cells we designed a novel form of constitutively active Akt1. Membrane attachment is a prerequisite for Akt to become activated by phosphorylation at threonine 308 and serine 473. In wild type Akt this recruitment to the membrane is mediated by binding of the PH domain to the membrane anchor PIP_3_ generated by the PI3K. The subsequent conformational change opens the catalytic domain and renders the kinase accessible for phosphorylation, which is a prerequisite for constitutive activation. Expression vectors for constitutively active Akt are described, that accomplish membrane insertion by an N-terminal myristylation tag that attaches the protein to the membrane (Franke *et al*, 1997). However, protein myristylation is a covalent process and membrane insertion is irreversible due to the lack of enzymes capable of cleaving the protein myristylation. To avoid potentially hampered membrane dislocation in a constitutively active derivative of Akt, we designed an expression vector that encodes for human Akt1 devoid of its PH domain and fused with an C-terminal farnesylation sequence linked to a polybasic tail (Resh, 1994). A schematic representation this constitutively active farnesylated form of Akt1 is shown in Figure 1

.

### Functional characterisation of ectopically expressed farnesylated Akt1

**Figure 2:**
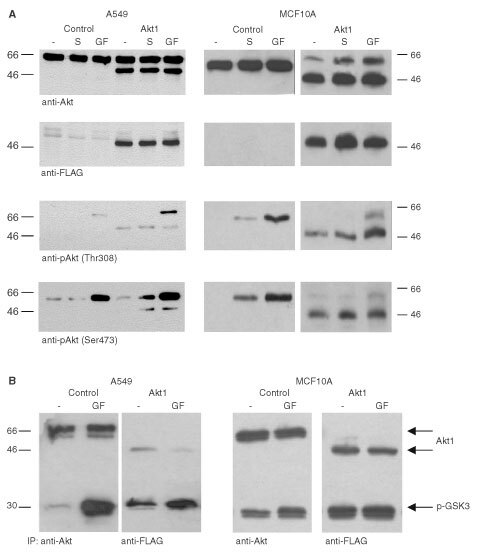
(**A**) Expression and constitutive activation of farnesylated Akt1 in A549 NSCLC cells and MCF10A mammary epithelial cells. Control transfected A549 and MCF10A cells, respectively or cells expressing farnesylated Akt1 (as indicated) were serum starved overnight and then stimulated for 15 min with medium containing 10% FCS (S) or 10% FCS plus a cocktail of 10 ng ml^−1^ each EGF, IGF-1, and PDGF (GF). Cell lysates were analysed by immunoblotting with antibodies specific for Akt1, FLAG-epitope, phospho-Akt (Thr 308) or phospho-Akt (Ser 473) as indicated. The ectopically expressed farnesylated Akt1 devoid of the PH domain migrates at a M_r_ of approximately 50 kDa. (**B**) Kinase activity of endogenous and ectopically expressed farnesylated Akt. Cells were serum starved for 16 h and then stimulated as described above. Immunoprecipitates of endogenous Akt from control transfected cells and farnesylated Akt from transfected cells were used in kinase assay reactions with GSK-3-fusion protein as substrate. Immunoblots were assayed with antibodies specific for phospho-GSK (Ser 21/9) (lower portion) and Akt (upper portion). Positions of phosphorylated GSK-fusion protein and Akt1 are indicated by an arrow.

MCF10A human mammary epithelial cells and A549 human NSCLC cells were stably transfected with the expression vector encoding farnesylated Akt1 devoid of its PH domain. Clones that were tested positive for ectopic expression of Akt1 by reactivity of the lysates with the M2 anti-FLAG antibody (MCF10A-Akt1 or A549-Akt1) were used in this study. To analyse whether the membrane targeting by a farnesylation tag renders ectopically expressed Akt1 constitutively active, the phosphorylation status of threonine 308 and serine 473 was determined in transfected cells as a marker of kinase activation (Alessi and Cohen, 1998). MCF10A-Akt1 and A549-Akt1 cells were seeded out in growth medium. After attachment the cells were serum starved for 16 h and then stimulated with medium containing 10% serum or 10% serum plus growth factors (EGF, PDGF, and IGF-1). Lysates were then analysed in immunoblot assays with antibodies specific for phospho-Akt (Thr 308) or phospho-Akt (Ser 473). Cells transfected with the empty vector were used as controls. The results are shown in Figure 2A

: while endogenous Akt was detected as a protein migrating at a M_r_ around 66 kDa, ectopically expressed Akt was detected with both, anti-Akt1 anti-FLAG antibodies as a protein with an obvious M_r_ of about 50 kDa. Endogenous Akt1 phosphorylation at Thr 308 and Ser 473 was only detectable after stimulation of the cells with a growth factor cocktail and, to a lesser extent, with serum. In contrast, farnesylated Akt1 was phosphorylated irrespective of serum conditions or growth factor stimulation.

To determine the kinase activity of endogenous and of ectopically expressed farnesylated Akt1 in MCF10A and A549 cells we performed an *in vitro* kinase assay with GSK-3-fusion protein as substrate. After attachment cells were serum starved for 16 h and then either kept untreated or were stimulated with medium containing 10% serum plus growth factors (EGF, PDGF, and IGF-1). Akt kinase activity was determined in an *in vitro* kinase assay as described in Materials and Methods. The results are shown in Figure 2B: in MCF10A cells endogenous Akt displayed a considerably lower kinase activity under low serum conditions as compared to conditions after stimulation with 10% serum and growth factors. Ectopically expressed farnesylated Akt1 showed comparable kinase activity under both stimulation conditions. Similar results were obtained in A549 cells. However, in these cells, the kinase activity of farnesylated Akt could still be enhanced by short incubation with 10% FCS and the growth factor cocktail. The results from the *in vitro* kinase assays and from phospho-Akt specific analysis show that removal of the PH domain and fusion of a farnesylation sequence to the C-terminus suffices to activate ectopically expressed Akt1 to obtain high levels of kinase activity.

### Suppression of anoikis upon expression of farnesylated Akt1

Untransformed cells commonly undergo programmed cell death after detachment from the extracellular matrix. This type of apoptosis has been termed anoikis (Frisch and Francis, 1994). Tumour cells must have evolved strong mechanisms for the suppression of anoikis in order to disintegrate from their original cellular environment, to extravasate and to form distant metastases. As matrix binding to cell surface integrins normally transmits a cell survival signal through the activation of the PI3K (Khwaja *et al*, 1997), we analysed whether expression of farnesylated Akt1 is able to suppress anoikis in MCF10A immortalised mammary epithelial cells.

**Figure 3:**
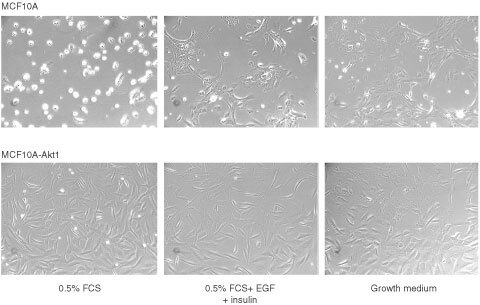
Suppression of anoikis in MFC10A cells expressing constitutively active Akt1. MCF10A control cells (upper panel) and MCF10A-Akt1 cells were seeded in 6-well plates in 3 ml of medium containing 0.5% horse serum (left), 0.5% horse serum plus EGF and insulin (middle panel), or normal growth medium (right panel). Microscopic pictures were taken 24 h post seeding.

The cells were seeded in high volume (3 ml) in 6-well plates in order to provide conditions that prevent the cell attachment before the onset of anoikis. Twenty-four hours later the cells were analysed microscopically and pictures were taken under the different medium conditions. The results are shown in Figure 3

. Cultivation of control transfected MCF10A cells (upper panel) in medium containing 0.5% serum without other supplements led to the onset of phenotypic markers of anoikis (left). MCF10A-Akt1 cells (lower panel) however, did not undergo anoikis under these medium conditions (left). Addition of insulin and EGF (middle column) or full medium (right column) suppressed the characteristics of anoikis in both, control transfected, and MCF10A-Akt1 cells.

**Figure 4:**
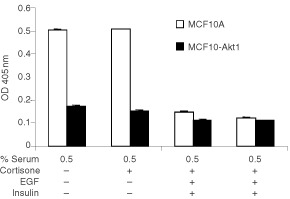
Suppression of anoikis in MFC10A cells expressing constitutively active Akt1. MCF10A control cells and MCF10A-Akt1 cells were seeded in poly-HEMA coated plates to prevent cell attachment under medium conditions as indicated in the figure. Sixteen hours later the cells were harvested, lysed and cell extracts analysed with an apoptosis ELISA kit according to the manufacturer's instructions. Results are mean±s.d. from three values.

An apoptosis ELISA that detects cytoplasmic histone-associated DNA fragments was performed to confirm that the phenotypic markers of anoikis coincide with molecular markers of apoptosis. Culture plates were pre-coated with poly-HEMA to prevent cell attachment. Control transfected MCF10A cells and MCF10A-Akt1 cells were cultured in coated plates under medium conditions as shown in Figure 4

and subsequently analysed for molecular features of apoptosis. Control transfected MCF10A cells were found to undergo apoptosis in poly-HEMA coated plates if grown in medium containing low serum without supplement of growth factors. Apoptosis could only be prevented by the addition of insulin or EGF, or full medium. MCF10A-Akt1 cells in contrast were resistant towards apoptosis under any of the conditions chosen in this experiment. The experiments were confirmed with two independent clones. The data from these experiments show that MCF10A cells still harbour the ability to undergo apoptosis upon loss of matrix detachment under the appropriate conditions. Expression of constitutively active farnesylated Akt1 alone is sufficient to efficiently suppress anoikis. Moreover, these results indicate that farnesylated Akt1 not only is phosphorylated at the positions described to be mandatory for full activity, but also exerts biological function when stably transfected in mammalian cells.

### Chemoresistance of A549 human lung cancer cells expressing farnesylated Akt1

**Figure 5:**
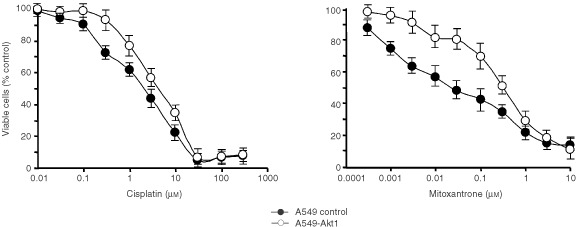
Chemoresistance of A549 human lung cancer cells expressing farnesylated Akt1. Control transfected A549 cells or A549-Akt1 cells were seeded in 96-well plates in medium containing 0.5% serum. Twenty-four hours later the cells were treated with different concentrations of cisplatin (left) or mitoxantrone (right) as indicated and incubated for another 72 h at 37°C. Cell viability was then determined with a standard XTT assay as described in Materials and Methods. Values are the mean of at least three independent experiments.

Intrinsic or acquired resistance towards chemotherapy is the most important limitation of current treatment options in cancer. While active transport mechanisms have been described to shuffle chemotherapeutic agents out of cells, other mechanisms are suggested that effectively suppress apoptotic pathways in tumour cells. Although Akt has been described in the suppression of a variety of apoptotic stimuli, relatively little is known about the modulation of chemosensitivity by Akt1. We therefore compared the chemosensitivity of control transfected A549 human NSCLC cells *vs* A549 cells expressing farnesylated Akt1 towards a regimen of clinically applied chemotherapeutics. The viability of the cells after incubation with the substances for 72 h was determined with a standard XTT assay as described in Materials and Methods. A549 cells expressing constitutively active Akt1 displayed an approximately 10-fold decreased sensitivity towards the DNA alkylating agent Mitoxantrone as compared to control cells (IC_50_ 0.2 μM *vs* 0.02 μM, respectively). A more than two-fold decreased sensitivity was observed upon treatment of the cells with the DNA alkylating agent cis-platin (Figure 5

). Interestingly, no altered chemosensitivity was observed with the M-phase specific tubulin inhibitor paclitaxel. A three-fold decreased sensitivity of A549-Akt1 cells was observed towards 5-fluorouracil. However, the cytotoxicity even at the highest concentrations did not exceed 70% of untreated control cells. It is noteworthy to mention that a differential chemoresistance was only observed when the cells were cultured in medium containing low serum. Under normal serum conditions the activation of endogeneous Akt probably suffices to render both cell lines more chemoresistant irrespective of ectopic expression of constitutively active Akt.

The IC_50_ values towards the various chemotherapeutics are summarised in Table 1

**Table 1:**
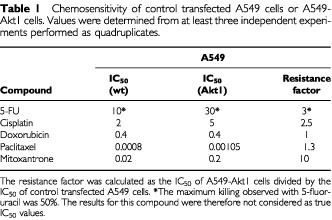
Chemosensitivity of control transfected A549 cells or A549-Akt1 cells. Values were determined from at least three independent experiments performed as quadruplicates

. No difference was observed upon treatment with doxorubicin. At lower doses the anthracyclin doxorubicin has been described as an inhibitor of DNA topoisomerase 1, while at higher concentrations it can intercalate with DNA. Interestingly, in MCF10A cells expressing farnesylated Akt1 we found a marked decrease in sensitivity towards both, cisplatin and doxorubicin, but consistently not towards paclitaxel (data not shown).

From these data we conclude, that the expression of activated Akt1 in A549 human NSCLC cells can cause partial resistance towards a defined class of chemotherapeutics, namely DNA reactive agents, but in our system does not alter the sensitivity towards cell cycle (M-phase) specific chemotherapeutics like paclitaxel.

## DISCUSSION

In this study, we investigated the influence of constitutively activated PKB/Akt1 on the onset of apoptosis in MCF10A immortalised mammary epithelial cells as measured by protection from anoikis and on the chemosensitivity of A549 human NSCLC cells. For these studies we utilized an expression vector encoding Akt1 devoid of its PH domain and a C-terminally fusion of a farnesylation sequence. Activation of farnesylated Akt was determined by analysing the phosphorylation status of the ectopically expressed Akt at Thr308 and Ser473 as well as by *in vitro* kinase assays. While we observed phosphorylation of the farnesylated Akt at both sites in MCF10A cells under low serum conditions, the phosphorylation of these amino acids was less well pronounced in A549 cells. However, immunoprecipitated farnesylated Akt was shown in *in vitro* kinase assays to be highly active in both cell lines. Surprisingly, the kinase activity of farnesylated Akt in A549 cells could further be stimulated by growth factors. We explain this by the fact that activation of Akt requires the phosphorylation at Thr308 by PDK1. In these cells, the activity of PDK1, which can be stimulated by growth factors through the generation of phosphoinositol trisphosphates (Currie *et al*, 1999) may be the rate limiting step for activation of both, endogenous and ectopically expressed Akt.

We were able to demonstrate that farnesylated Akt1 was a very potent inhibitor of anoikis in MCF10A immortalised human mammary epithelial cells. We selected this cell line, since it still retains the capacity to undergo apoptosis upon loss of substrate attachment. In this context we investigated the involvement of focal adhesion kinase (FAK) in the suppression of anoikis (Frisch *et al*, 1996). However FAK was not found to be phosphorylated or active to a higher degree in MCF10A-Akt1 cells as compared to control cells (data not shown). We therefore assume that enhanced FAK activity does not play a major role in the prevention of anoikis in this cellular system. Our results with MCF10A cells are in agreement with data from Rytömaa *et al* (2000) who were able to prevent anoikis of early passages of Madin-Darby canine kidney cells (MDCK) by activated ras (V12 mutation) or PKB. These data together with our chemosensitisation studies indicate, that Akt may have a dual role in tumorigenesis: Early in the process of malignant transformation Akt may prevent anoikis when immortalised cells leave their original cellular context to extravasate into a different environment and later to protect cancer cells from unfavourable conditions of e.g. poor nutrient supply and low oxygen to transmit strong survival signals. The clinical consequence finally would be reflected in a significantly reduced chemosensitivity of cancers with an activated Akt pathway. However, it has to be emphasised that multiple factors such as overexpression of multidrug transporter proteins, host derived factors or other failures in the apoptotic machinery (e.g. loss of caspase or Apaf-1 expression (Soengas *et al*, 2001; Tannock, 2001) significantly contribute to the frequent failure of chemotherapy in the clinic.

Surprisingly, although a wealth of information exists describing the role of Akt in conveying survival signals, only relatively few data are published describing an association of Akt activation and/or overexpression with chemoresistance. Page *et al* (2000) observed a 30% decreased sensitivity of Caov-3 human ovarian cancer cells expressing activated Akt at 10 μM paclitaxel. Mechanistically they explained their observation by an inhibition of cytochrome *c* release from mitochondria of cells expressing activated Akt. Yuan *et al* (2000) observed elevated Akt2 activity in 36% of analysed human ovarian cancer specimen. The majority of tumours with elevated Akt activity were graded stage III and IV. Interestingly, high PI3K levels did not necessarily correlate with Akt activation. This observation could explain the data of Mitsuuchi *et al* (2000), who did not observe altered drug sensitivity towards cisplatin and paclitaxel upon inhibition of the PI3K pathway by LY294002 in A2780 ovarian cancer cells. Another indirect link to the involvement of Akt in tumorigenesis stems from the observation that the tumour suppressor PTEN/MMAC1 is a direct counteractor of Akt activation by dephosphorylating the 3′ position of the membrane anchor PI 3,4,5-trisphosphate (Stambolic *et al*, 1998).

In this study we compared the sensitivity of A549 cells expressing constitutively active Akt1 towards five different chemotherapeutic agents. A profound desensitisation in cells expressing activated Akt1 as compared to control transfected cells was observed with Mitoxantrone and cisplatin. Similar data have also been obtained with MCF10A cells (data not shown). Both of these agents can exert their cytotoxic mode of action by inducing DNA damage irrespective of the cell cycle stage. Mitoxantrone has been described to intercalate with DNA, but also to target Topoisomerase II and to interrupt its catalytic cycle thereby leading to persistant double strand breaks of DNA (Boland *et al*, 2000). Furthermore, Mitoxantrone treatment of cells has also been shown to induce the transcription factor NF-κB, which then initiates the transcription of several anti-apoptotic genes and also of the gene for the inhibitor of κB kinase (IKK) (Engels *et al*, 2000). A possible mechanism for chemoresistance towards Mitoxantrone mediated by constitutively active Akt might be the phosphorylation of IKK by Akt, which enhances the degradation of IκB and consequently the translocation of NF-κB into the nucleus (Ozes *et al*, 1999). Cisplatin has been described to lead to activation of Caspase 8 with subsequent initiation of the proteolytic caspase cascade (Micheau *et al*, 1999a,b; Engels *et al*, 2000). A possible mechanism for the chemoresistance mediated by constitutively active Akt might be a down-regulation of FADD-Protein or the upregulation of the caspase 8 inhibitor c-FLIP (Panka *et al*, 2001). In contrast, no modulation of chemosensitivity was seen with chemotherapeutic agents that function only in a specific phase of the cell cycle, like paclitaxel that only targets cells in mitosis. Paclitaxel differs from cisplatin or Mitoxantrone in its mechanism of action. Unlike cisplatin or Mitoxantrone, that target the DNA and induce strong DNA damage responses, paclitaxel is a spindle poison that induces a mitotic arrest by excessively stabilising the microtubules (Crossin and Carney, 1981) responsible for chromosomal segregation. No pathways are described to date in which Akt1 can interfere to prevent the induction of apoptosis after disruption of the spindle apparatus with subsequent mitotic arrest. Doxorubicin at lower concentrations acts as a topoisomerase II-inhibitor and thus exerts its mechanism of action in a cell cycle-dependent manner. At higher concentrations Doxorubicin intercalates with the DNA leading to strand breaks in a non cell cycle-dependent fashion (Ross and Bradley, 1981; Bodley *et al*, 1989). Thus, the cytotoxicity of Doxorubicin at lower concentrations is cell cycle dependent, while at higher concentrations it can also kill cells that are not actively progressing through the cell cycle (Schmidt *et al*, 2001). It will be interesting to study in detail the molecular mechanisms downstream of Akt1 that mediate chemoresistance towards Mitoxantrone and cisplatin.

In summary, we could show that the expression of an activated form of Akt1 can confer resistance of immortalised epithelial cells towards apoptosis induced by loss of substrate attachment and can induce chemoresistance towards certain classes of chemotherapeutic agents like cisplatin or Mitoxantrone. These data further emphasise that Akt1 may be a well suited target for directed tumour therapy at both, early and advanced stages of tumorigenesis to prevent spreading of the disease and to resensitise established tumours for standard chemotherapy.
